# The unseen costs of medical training in the UK: a growing crisis

**DOI:** 10.3389/fmed.2025.1592010

**Published:** 2025-05-14

**Authors:** Waseem Jerjes, Azeem Majeed

**Affiliations:** Faculty of Medicine, Imperial College London, London, United Kingdom

**Keywords:** financial stress, postgraduate training, hidden costs, workforce sustainability, burnout, specialty disparities, patient safety, structural reform

## The silent financial strain of medical training

Medical training in the United Kingdom is renowned for being highly regarded and thoroughly preparing physicians. There is, however, a vast and largely covert economic burden that underlies the prestigious pathway, which trainee doctors must endure. Tuition fees and living costs are highly recognized, but there are numerous covert costs to medical training that accumulate over time, inducing severe economic and emotional strain on students and junior doctors. These covert costs extend to individual distress, impacting morale in the workforce, levels of retention, and the system's wider viability.

The level to which this issue is impacting the medical community is only more so in recent times. Medical students in the UK graduate with record debts, with individuals accumulating liabilities as much as £100,000 by the time they graduate. The economic pressure is carried over into postgraduate training, where compulsory costs such as professional examinations, specialty membership, and additional training requirements contribute to the economic pressure. An example is the situation with surgical trainees, where they could have to spend as much as £70,000 to fulfill the requirements to qualify, putting a lot of economic pressure on them when their income is still limited. These figures point to the rising disparity in medical training and point to the urgent need to reform so that economic restrictions do not control career progression and deter individuals from entering the field (1).

This view point addresses the hidden economic costs in medical training, their pervasive impacts on trainee learning, health, and professional development. Furthermore, it considers the broader implications on patient outcomes and the performance of the health system. In so doing, we aim to encourage system reforms that reduce economic distress to later medical graduates, toward a more equitable and sustainable training system.

## The financial burden on trainees: a system designed for the privileged?

Medical training in the United Kingdom is an expensive proposition for would-be physicians, and it raises concerns about equitable access to the medical profession. Tuition fees are an accepted expense, but other associated costs related to professional examinations, moving house, and mandatory membership are disproportionately borne by individuals from lower socioeconomic backgrounds. These hidden costs are more than just fees, and they have an effect on career choice, welfare, and staff viability in the National Health Service (NHS).

Postgraduate medical training is a series of high-stakes examinations, specialty courses, and certifications, all requiring large economic expenditures. Cross-sectional analysis revealed that a UK trainee will spend £9,105 on course fees, £5,411 on conference fees, and £4,185 on examination fees—a total of an estimated £18,701 in unreimbursed costs during training (Table 1). The costs are even higher in highly specialist specialties where the total can mount to £71,431 (2). These costs indicate the disproportionate economic burden placed on trainees, most of whom must fund their way through training on salaries that do not meet these expenses.

**Table 1:** The hidden financial costs of medical training in the UK—A breakdown across stages and specialties.

**Expense category**	**Estimated cost (£)**	**Notes (impact and variation)**
Undergraduate tuition fees	£9,250 per year (UK) £30,000+ per year (international)	UK students complete medical school with debts up to £100,000, while international students face even higher financial burdens.
Undergraduate maintenance loans and living Expenses	£8,000–£15,000 per year	Cost of living varies by location (London is significantly higher). Many students rely on additional parental support or part-time work.
Postgraduate training costs	£15,000–£71,000+	Specialty-dependent. Higher for surgical and procedural specialties due to exam fees, courses, and equipment costs.
Postgraduate exam fees (MRCP, MRCGP, MRCS, FRCA, etc.)	£4,000–£18,000	Higher for competitive specialties requiring multiple exams (e.g., MRCS Part A and B: £2,300+ per attempt). Resits add further costs.
Mandatory training courses (ALS, ATLS, BSS, PLS, etc.)	£2,000–£10,000	Essential for career progression but rarely reimbursed. Some specialties require multiple expensive courses.
Conference fees and presentations	£500–£2,500 per event	Expected for career advancement. Often self-funded, despite being a requirement for specialty training applications.
Relocation costs (multiple moves across training)	£1,500–£5,000 per move	Junior doctors move every 6–12 months, with costs including deposits, rent advances, moving fees, and commuting expenses. London-based trainees face the highest rents.
Professional membership fees (GMC, Royal Colleges, MDU, MPS, BMA, etc.)	£420–£3,000 per year	Non-negotiable costs to maintain registration and medical indemnity. Some specialties (e.g., surgeons, anesthetists) have higher indemnity costs.
Medical indemnity insurance	£500–£10,000 per year	Indemnity costs skyrocket for independent work (e.g., private practice, procedural specialties). Junior doctors doing extra shifts face additional coverage costs.
Equipment and specialty-specific tools	£500–£5,000+	Surgeons and dentists require expensive personal equipment not covered by NHS employers.
GP and specialist registration fees	£2,000 - £3,500	Final registration costs after passing specialty exams. GP trainees face MRCGP fees, while consultants require CCT registration payments.
Income gap during transition to specialty training	Loss of 3–6 months of salary	Many doctors experience a period of unpaid time between training rotations, causing significant financial instability.
Cost of working locum shifts to cover expenses	Earned income used to offset costs	Many trainees take on additional shifts, but this leads to burnout and reduced study time, worsening mental health and training outcomes.

One major economic challenge is the frequent relocations that are part of medical training. Trainees will have to make multiple relocations between regions to complete clinical attachments, and with them come multiple costs related to moving, housing deposits, and varying living expenses. In other fields where relocation is typically discretionary or paid by the employer, junior doctors must make these relocations as part of their career development, with little economic assistance. While there are no accurate relocation costs available, these multiple economic outlays represent most of the economic costs of medical training.

In addition to direct training costs, professional status in medicine also carries recurrent costs. All doctors must have annual GMC registration, and fees add to the overall cost of being in the profession. Medical indemnity cover is also needed to work clinically, and fees are charged by specialty and level of training. Resident doctors have been estimated to spend between £420 and £3,000 per year on professional fees (3). These costs, though necessary to maintaining high standards in patient care, contribute to trainee stress, particularly from financially disadvantaged backgrounds.

The cumulative effect of these costs is important in relation to the diversity and inclusivity of the medical profession. There have been moves to widen access to medicine, but only 5% of students coming into UK medical school are from working-class backgrounds, in contrast to 75% from higher socioeconomic backgrounds (4). The expense of training may deter able individuals from coming into medicine or motivate individuals from lower socioeconomic backgrounds to take on excessive debts to fund their studies. This is an uneven playing field where economic security, and not ability or clinical promise, is a determining factor in medical training.

Besides economic hardship, the economic stress of medical training exacts a real cost on trainee wellbeing. The British Medical Association (BMA) survey revealed that six out of every 10 UK medical students reduced or ceased to spend on essentials due to economic pressures (5). In extreme cases, economic issues lead others to give up their career choice altogether—a study revealed that four out of every 10 medical students had seriously thought about withdrawing from medical school due to economic pressures (6).

The funding challenges facing medical training are not only a problem for individual trainees but have broader implications for the NHS as an institution. The increased economic pressure being put on medical trainees is set to increase existing shortages within the NHS, particularly in underfunded and underprivileged areas where there is already an entrenched problem in recruiting. If economic uncertainty is to continue to discourage high-caliber individuals from entering or completing medical training, the long-term sustainability of the NHS workforce will be placed under pressure, ultimately affecting patient care delivery.

These funding challenges are not unique to the UK or high-income nations. Emerging economies like Brazil, China, and India are also experiencing considerable strain in medical training due to rapid expansion, limited state support, and growing urban–rural disparities. In Brazil, decentralized medical education expansion has led to concerns about inadequate support structures and financial pressure on students from rural backgrounds (7). In China, reforms in medical education have intensified the academic burden without equivalent financial assistance, contributing to burnout among medical interns (8, 9). In India, the high cost of private medical education, compounded by limited postgraduate training seats, results in both financial hardship and emigration (10). Including these global perspectives underlines that the financial pressures in medical training are part of a wider, systemic issue that transcends borders and income levels.

## How financial strain affects training and career development

Financial pressure is one major driver in determining the academic and professional progression of medical trainees, specialty choice, their mental health, and the healthcare system as a whole. The increased costs of postgraduate and medical training discourage trainees from pursuing less financially rewarding specialties, leading to shortages in key specialties. In particular, economic costs associated with pursuing certain specialties such as infectious diseases discourage many aspiring doctors from pursuing these specialties. Northwestern University's Feinberg School of Medicine pediatrics professor, Dr. Tina Tan, cited the persistent issue in recruiting trainees into infectious diseases due to the imbalance in compensation and economic feasibility. The effect is most visible in the United States, where over 80% of counties lack an infectious disease specialist. High costs related to medical training, and relatively lower compensation in specialties such as infectious diseases, psychiatry, and general practice, present a disincentive to trainees to pursue these critical but less lucrative specialties (11).

The economic expense of medical training is not confined to specialty selection, and it highly affects the trainees' mental wellbeing. Financial stress affects both mental health and satisfaction with career progression. One stark demonstration of the influence of economic and system pressure on health workers is in New South Wales, Australia, where over 120 staff specialist psychiatrists resigned their positions due to concerns over unworkable workloads, poor remuneration, and patient safety. This is a sign of the far-reaching effects of economic pressure, not only on individual workers, but on the stability of health services (12).

Financial pressures also have significant implications in terms of the quality of training. Where resources are scarce and trainees are overworked or have to work extra jobs to help subsidize the costs of training, their ability to focus on learning and development is compromised. One dramatic manifestation of this tension was witnessed in South Korea, where a 13-month standoff occurred over government proposals to increase 2,000 places in medical school admissions. The initiative, which was meant to address an anticipated doctor shortfall by 2035, was opposed by trainee doctors, who perceived it would undermine the quality of medical training rather than address the structural issues in key care specialties. The case illustrates the tension between expanding the workforce, economic pressure, and training quality (13).

The economic strain of medical training in the long-term can also hinder professional development. In the United States, the costs of medical training and the scarcity of funded positions have been key factors in an impending physician deficit, particularly in rural medicine and primary care. The average US medical graduate finishes training owing around $200,000, an economic strain that drives career choice and diverts trainees from underfunded but essential specialties. The economic barriers to entering and remaining in medical training thus play a definitive role in determining the healthcare landscape and maintaining present shortages in key specialties (14).

In the UK, parallel economic pressures have contributed to longstanding challenges to retaining junior doctors in the NHS. Workforce shortages have remained a persistent problem, coupled with an increased reliance on internationally qualified medical staff and an imbalance between domestic production of medical school graduates and postgraduate training places. In 2023, over two-thirds of new recruits to the NHS were non-UK graduates, a sharp rise from under half in 2017. While internationally qualified doctors have filled the gap in the workforce, they have also served to highlight inefficiency in domestic workforce planning. Conservative policies to raise domestic medical school places have inadvertently created postgraduate training bottlenecks, leading to lost potential as UK-trained graduates cannot secure training posts. The ensuing disillusionment between junior doctors, coupled with economic pressure, has led to demotivation and increased levels of attrition, with junior doctors choosing to pursue more financially viable options elsewhere (15).

The combined result of finance pressure, weak workforce planning, and system inefficiency places medical trainees in an increasingly precarious position. Unless there is significant change, the impacts will extend to patient care, workforce sustainability, and the stability of the system as a whole. Addressing these issues requires a system-wide transformation in how medical training is funded, how postgraduate training is planned, and how finance is tied to incentives and to the needs of the workforce.

## The patient perspective: how financial stress affects patient care

Financial stress among medical trainees and healthcare professionals has far-ranging consequences, one of the most severely impacted being patient care. Financial uncertainty forces the majority of trainees to work overtime or take part-time jobs, leading to physical and emotional exhaustion. Overtime to meet economic pressure can compromise attention, delay critical decisions, and increase the chances of clinical errors.

Evidence consistently links economic pressure to mental health decline among medical trainees. Greater levels of debt and economic uncertainty have been linked to depression, anxiety, and insomnia. One study on the effects of economic stress on clinical psychology graduate students found that economic hardship resulted in delayed major decisions and even foregone required health care, with 17.5% experiencing a medical emergency they could not afford. The same study found that economic stressors led to increased worrying time, poorer quality sleep, and poorer general wellbeing (16).

The effects of financial stress do not only influence individual wellbeing but also have an effect on training performance and professional development. High debt levels adversely affect academic performance, mental health, and drive specialty choice toward more lucrative fields. This financially driven choice affects the delivery of healthcare services in the long run, particularly in primary care, where shortages have been an ongoing problem (17).

The direct linkage between patient safety and economic stress cannot be ignored. Burnout, associated with economic stress, is linked to reduced patient satisfaction, higher likelihood of diagnostic errors, and compromised clinical decision-making. Economically stressed healthcare providers have been found to demonstrate reduced empathy, emotional exhaustion, and work disengagement. One study assessing the wellbeing of medical staff found that higher economic stress was associated with reduced job satisfaction and mental health. This resulted in reduced patient engagement, lower attentiveness, and higher likelihood of medical errors (18). The interaction between economic pressure and mental exhaustion places both healthcare providers and their patients in a vulnerable position, putting them at risk of adverse outcomes in clinical practice.

Addressing financial stress in medical trainees is not just an economic or workforce issue—it is a key patient safety concern. Interventions to minimize financial stress must be embedded in medical education and workforce planning. Financial literacy training could give trainees the tools to manage debt, budget for the future, and reduce stress. In addition, formal systems, such as interest-free loans or means-tested bursaries, could help to minimize the immediate pressures leading to working long hours and burnout. Investment in trainee welfare is not only good for individual physicians, but is also necessary to preserve high standards in patient care. Medical schools and policymakers must understand that by alleviating the financial pressure on trainees, they are helping to contribute directly to a safer, more effective healthcare system (19).

## The NHS and beyond: why this is a national healthcare crisis

Financial stress in trainee doctors and healthcare staff is a rising issue in the NHS and the wider healthcare system. Financial pressure on trainees affects not only their personal wellbeing but also system-wide, contributing to undermining workforce sustainability, patient safety, and the efficiency of healthcare delivery.

The impacts on junior doctors and medical students are well described, with raised stress associated with somatic illness, drug abuse, and the probability of dropping out of medical studies before graduation or seeking other career alternatives to clinical practice. For those staying, the ongoing pressure to finance training, loan repayment, and mandatory professional costs is a chronic source of stress and dissatisfaction, damaging morale and long-term career continuity (20).

Besides individual wellness, economic stress is another major contributor to physician burnout. Burnout—marked by exhaustion, detachment, and low accomplishment, is common in the medical community, with rates over 50% in trainee and practicing physicians. Junior doctors' persistent economic stress, together with overwork and inadequate support systems, also contribute to increased burnout rates. This results in severe implications to the delivery of healthcare, as burnt-out physicians are more prone to cognitive overload, depersonalized patient relations, and errors in clinical judgment. The resulting decline in the quality of care imposes more pressure on an already stretched healthcare system, increasing healthcare costs and reducing work force efficiency (21).

Financial strain and overwork raise the risk of clinical errors. In England, there are 237 million errors in medication annually, and 1,700 deaths as a consequence, which costs the NHS over £98 million annually. Furthermore, a UK-based study concluded that the NHS costs £14.7 billion annually to treat patients harmed by errors in care, and 820 avoidable deaths are caused by systemic patient safety failings. These findings indicate the need for structural solutions to financially and psychologically support medical workers, reducing stress-related errors and generally improving outcomes in care (22).

Staff shortages are another issue driven by budget pressures. The NHS is still finding it hard to attract and retain doctors in all specialties, but most significantly in general practice, emergency medicine, and psychiatry. Financial pressure discourages trainees from pursuing these valuable but underfunded specialties, compounding already-present gaps in services. At the same time, economic uncertainty faced by junior doctors makes career choices in better-funded healthcare systems elsewhere more attractive, and so there is an exodus of UK-trained doctors. The chronic loss of highly qualified medical staff imposes pressure on remaining staff, leading to a cycle of stress, reduced workforce capacity, and poor-quality services. Unless steps are taken to minimize financial barriers to medical training and practice, the NHS is facing an accelerating workforce crisis that could have long-term consequences for patient care and the delivery of services.

Policymakers, healthcare organizations, and professional groups have to make addressing financial stress in medical trainees and doctors a priority. Root-and-branch reforms have to be introduced to avoid financial insecurity continually harming morale, jeopardizing patient safety, and destabilizing the NHS. Investment in formal financial assistance, sustainable workforce planning, and targeted retention is the key to maintaining a strong and effective healthcare system. Unless there is collective action, the strain on medical professionals' finances will keep growing, fuelling shortages and compromising the quality and sustainability of patient care.

## Solutions: a call for structural reform

An adequate holistic response to the UK trainee doctors' financial pressures must address the immediate and structural challenges on all fronts (23). The current funding and workforce models do not take into account the long-term implications of economic pressure on retention, patient safety, and NHS stability. The problem is not just the cost of training but how these economic barriers reinforce inequalities, drive shortages, and deter potential and current talent from entering or remaining in the profession. Reform must reach farther than modest budget adjustments, embedding sustainable and visionary policies that treat funding as an investment, not a cost. The healthcare workforce is counting on it (Table 2).

**Table 2:** A multi-level reform strategy to alleviate the financial burden on medical trainees.

**Proposed reform**	**Implementation strategy**	**Expected impact**	**Feasibility and barriers to overcome**
Postgraduate training fund (loan scheme for specialty training)	Government-backed structured loan scheme for postgraduate trainees, with repayment tied to salary bands (similar to undergraduate loans but specific to doctors).	- Reduces financial stress for trainees - Prevents reliance on high-interest loans - Encourages equal access to specialty training	Feasibility: high (requires NHS and Treasury coordination) Barriers: political resistance to upfront funding
Exam fee subsidization and capped resit costs	NHS/Health Education England (HEE) subsidizes specialty exams, with a limit on resit fees after two failed attempts.	- Prevents financial penalty for failing once - Encourages focus on training rather than financial anxiety	Feasibility: medium (requires funding allocation) Barriers: professional bodies (e.g., Royal Colleges) may resist fee reductions
Service-linked training bursaries for underfilled specialties	Expansion of bursaries for GPs, emergency medicine, psychiatry, and geriatrics, tied to minimum 3-year NHS service post-qualification.	- Directly addresses workforce shortages - Reduces reliance on international recruitment - Encourages doctors to remain in underfunded fields	Feasibility: high (similar schemes exist in military medicine and rural Australia) Barriers: retention enforcement; must ensure flexibility
NHS-sponsored accommodation for trainees in high-cost areas	NHS partners with local councils to provide subsidized housing near hospitals or regional hubs, with rental costs capped at 30% of salary.	- Reduces relocation stress - Prevents high rental costs affecting financial stability - Increases workforce retention in urban areas	Feasibility: medium (requires NHS/council coordination) Barriers: housing market constraints, limited initial funding
Employer-covered professional Fees (GMC, indemnity, royal college memberships)	NHS employers cover a portion or full cost of GMC registration, indemnity insurance, and professional memberships for trainees up to CCT level.	- Removes direct financial burden on trainees - Reduces disparity between specialties (surgical indemnity is much higher)	Feasibility: medium (requires NHS contractual changes) Barriers: resistance from medical defense unions
Flexible training pathways and paid fellowships to prevent burnout	NHS creates structured part-time pathways for trainees needing financial support, with additional paid research or teaching fellowships.	- Prevents over-reliance on locum work - Increases financial stability without affecting training quality	Feasibility: high (some specialties already offer this) Barriers: requires cultural shift toward flexible training
Regional training models to reduce unnecessary relocation	Medical training placements restructured to minimize mandatory relocations, with options for longer placements in regional hubs.	- Reduces moving costs - Increases continuity of training and patient care - Improves work-life balance for trainees	Feasibility: high (some pilot models exist) Barriers: requires regional NHS deanery cooperation
Government-backed tax relief for training costs	Doctors can claim tax relief on exam fees, training courses, and relocation expenses, similar to schemes for self-employed professionals.	- Immediate financial relief - Reduces out-of-pocket costs for self-funded training elements	Feasibility: medium (requires tax code reform) Barriers: treasury pushback on potential tax revenue loss
NHS-funded online training and virtual reality (VR) for skill development	Shift toward VR and simulation-based skill training (e.g., surgical techniques, resuscitation) to reduce expensive in-person course fees.	- Makes training more accessible - Reduces financial burden of repeated practical courses - Future-proofs medical education	Feasibility: high (technology is advancing rapidly) Barriers: requires NHS investment in simulation centers
National financial literacy programme for medical trainees	Mandatory financial planning modules in medical school and postgraduate training, covering debt management, pensions, and tax relief.	- Reduces long-term financial stress - Increases awareness of available financial support options	Feasibility: high (already done in other professional fields) Barriers: medical education curriculum restructuring required

Postgraduate training must be structurally reformed to become financially sustainable and accessible (24). Unlike undergraduate training, which provides a formal system of student loans, postgraduate training asks doctors to finance their development themselves, and so creates an uneven playing field. This results in a career where those with more resources have an advantage, and others are driven into debt or long hours, eventually damaging their training and health. Implementing a national postgraduate training fund, modeled on the undergraduate system but adapted to the realities of medical trainees, would bring in a formal system of funding that is repaid over time under more favorable terms than commercial loans. The system must be set so that doctors can complete training without undue financial stress, so that they can focus on learning clinical expertise and not merely how to survive the process.

Financial incentives must also be matched to the needs of the workforce (25). The present funding support systems fail to cover specialties and geographical locations with severe shortages. Supplementing bursary schemes linked to commitments to work in underfilled specialties can be an immediate solution to recruiting and retaining staff. Tuition fees, professional examinations, and other training costs should be covered by these bursaries in return for a commitment to work in the NHS in certain locations for an agreed number of years. This system has been used successfully in government-funded healthcare systems worldwide, and introducing it to the NHS could stabilize the workforce planning while, simultaneously, easing the economic pressure on trainees.

Housing and relocation remain two of the most important financial concerns for trainees, but nothing is being done to address the root causes of these costs. In place of modest relocation allowances that do not meet the real economic strain, the NHS should explore direct housing solutions for trainees. In city centers where housing is costly, purpose-built subsidized housing would significantly reduce economic pressure, improving junior doctors' quality of life and their likelihood to stay. In rural towns, subsidized long-term housing should be included in rural recruitment and retention strategies to attract and retain medical personnel to these towns. International healthcare systems, as in Australia and Canada, have already recognized housing support as a key investment in the workforce, and the UK must do the same to keep up.

Mandatory professional membership, indemnity cover, and course fees represent another expense (26). In contrast to other careers where these costs are paid by employers, trainee doctors are required to meet these out-of-pocket, accumulating thousands of pounds in extra costs during their training. The NHS needs to take more responsibility to meet these necessary costs, especially during the initial years of training, where pay is low and finances are tight. Professional indemnity, in particular, needs to be included in NHS job contracts instead of individual doctors having to organize cover themselves. Although there would be an initial expense, the long-term gains in better retention, less burnout, and more stable workforces would more than compensate.

Financial stress is not just about overt costs—it is also about the lack of long-term career development and financial literacy during medical training. Trainee doctors make poor decisions about finances as a result of poor knowledge about tax relief options, pension savings, and financial strategy. Financial literacy needs to be incorporated into medical training from an early point, equipping trainee doctors with the tools to deal with their income, investments, and long-term finances. High-stress professions such as law and finance have formalized financial planning tools in place to benefit employees, yet medical training continues to overlook this important area. Requiring compulsory financial learning modules would assist doctors in making better long-term financial decisions, reducing stress and boosting overall job satisfaction.

Workforce sustainability must also be reconsidered through more flexible working models that allow doctors to balance financial stability with their wellbeing (21). Burnout and financial stress are deeply interconnected, with many doctors taking on excessive locum shifts to manage debt, often at the expense of their own health. A more sustainable model would involve structured part-time training pathways with financial supplementation, allowing doctors to manage their financial obligations without resorting to excessive work. Additionally, expanding opportunities for paid research fellowships, teaching roles, and administrative positions within the NHS would create alternative income streams that contribute to professional development rather than merely serving as a financial lifeline.

The UK also needs to look outwards to adopt best practice from other nations' healthcare systems. In Australia and Canada, doctors are offered monetary incentives to work in poor areas for a set amount of time under long-term contract commitments. This ensures stability in the workforce and provides economic stability to doctors during their early years. Governments in European systems provide direct funding to train specialist doctors rather than requiring doctors to fund their own examinations and continuous learning. The UK should study these systems, modifying them to meet the specific requirements of NHS manpower planning and medical training.

In Australia, for example, the Rural Health Multidisciplinary Training Program provides housing subsidies, bonded scholarships, and structured training pathways to encourage doctors to remain in underserved regions. Similarly, Canada's Family Medicine Expansion initiative offers funded postgraduate slots with guaranteed practice placements in rural areas. In the U.S., the National Health Service Corps offers debt forgiveness in exchange for service in health professional shortage areas. These models demonstrate that aligning financial incentives with workforce needs is not only possible but already in practice globally—offering feasible templates for UK adaptation.

Technology can also reduce the economic strain on trainee medics. Repeated visits to revision courses, conferences, and practical training days are an expensive outlay. Transitioning toward NHS-funded online training modules would reduce costs without compromising standards. Training via virtual reality and simulation could reduce the need for expensive practical face-to-face courses, and increase access to and the viability of medical training.

While a comprehensive economic analysis is beyond the scope of this article, preliminary estimates suggest that many proposed interventions are financially viable. For instance, subsidizing exam fees and covering professional memberships for all UK trainees up to the Certificate of Completion of Training (CCT) could cost under £25 million annually. This figure is minimal compared to the £14.7 billion the NHS spends each year treating patients harmed by care mistakes. Similarly, investing in subsidized housing for trainees could reduce reliance on locum staff and improve retention rates. These reforms should be viewed not merely as expenses but as strategic investments aimed at enhancing workforce stability and reducing systemic inefficiencies (27).

Lastly, the funding challenges faced by medical trainees are more than a case of the cost of professional examinations or membership fees—they are symptoms of a broader failure to treat medical training as an investment in the healthcare system's future. Until the NHS and policymakers recognize that funding trainees is a strategy for the workforce, not a budget constraint, these problems will persist. There must be an attitude shift, away from short-term budget reductions and toward long-term investment in medical personnel. Doing nothing will have clear consequences: persistent shortages in the workforce, lower standards of patient care, and the migration to better-funded healthcare systems elsewhere of talented physicians. The solutions are there, but they will only come through political will and system reform. Reform must not only originate from medical training but must be driven by national healthcare policy, so that funding stability and workforce sustainability are part and parcel of the NHS's future.

Although this paper focuses on the NHS, the proposed structural reforms—such as subsidized training, regional retention strategies, and integrated housing policies—could be adapted to other universal healthcare systems. By reframing workforce investment as a global public good, countries at different stages of development can work toward resilient, equitable health systems.
